# Cellular Changes in Retinas From Patients With *BEST1* Mutations

**DOI:** 10.3389/fcell.2020.573330

**Published:** 2020-10-14

**Authors:** Vera L. Bonilha, Brent A. Bell, Meghan J. DeBenedictis, Stephanie A. Hagstrom, Gerald A. Fishman, Joe G. Hollyfield

**Affiliations:** ^1^Department of Ophthalmic Research, Cole Eye Institute, Cleveland Clinic, Cleveland, OH, United States; ^2^Department of Ophthalmology, Cleveland Clinic Lerner College of Medicine, Case Western Reserve University, Cleveland, OH, United States; ^3^Scheie Eye Institute, University of Pennsylvania, Philadelphia, PA, United States; ^4^Pangere Center at The Chicago Lighthouse for People Who Are Blind or Visually Impaired, Chicago, IL, United States

**Keywords:** best disease, *BEST1* gene, histopathology, retinal pigment epithelium, photoreceptors

## Abstract

Best disease (BD), also known as vitelliform macular dystrophy, is an inherited disease of the central retina caused by more than 300 pathogenic variants in the *BEST1* gene. The phenotype of BD is variable, and there are just a few reports on the histopathology of eyes from donors with BD. Here, we describe the histopathological comparison of donor’s eyes from two patients with BD. Eyes obtained from 85-year-old (donor 1) and 65-year-old (donor 2) donors were fixed within 25 h postmortem. Perifoveal and peripheral retinal regions were processed for histology and immunocytochemistry using retinal-specific and retinal pigment epithelium (RPE)-specific antibodies. Three age-matched normal eyes were used as controls. DNA was obtained from donor blood samples. Sequence analysis of the entire *BEST1* coding region was performed and identified a c.886A > C (p.Asn296His) variant in donor 1 and a c.602T > C (p.Ile201Thr) variant in donor 2; both mutations were heterozygous. Fundus examination showed that donor 1 displayed a macular lesion with considerable scarring while donor 2 displayed close to normal macular morphology. Our studies of histology and molecular pathology in the perifovea and periphery of these two BD donor eyes revealed panretinal abnormalities in both photoreceptors and RPE cellular levels in the periphery; donor 1 also displayed macular lesion. Our findings confirm the phenotypic variability of BD associated with *BEST1* variants.

## Introduction

Best vitelliform macular dystrophy is an inherited disease of the central retina caused by pathogenic variants in the *VMD2* gene, now known as *BEST1* (Marquardt et al., 1998; Petrukhin et al., 1998). More than 300 disease-causing variants in the *BEST1* gene have been reported (Johnson et al., 2017; Nachtigal et al., 2020). Pathogenic variants in this gene are linked to at least three distinct retinopathies that can be distinguished by phenotype and mode of inheritance: the autosomal dominant Best vitelliform macular dystrophy or Best disease (BD), the autosomal dominant vitreoretinochoroidopathy (ADVIRC), as well as the autosomal recessive bestrophinopathy (ARB) (Nachtigal et al., 2020). The *BEST1* gene encodes Bestrophin-1, a regulator of intracellular Ca^2+^ localized at the basolateral membrane of the retinal pigment epithelial (RPE) cells (Marmorstein et al., 2000).

The morphological findings described in the eyes of BD patients evaluated with spectral domain optical coherence tomography (SD-OCT) are variable and, in sum, include (1) the accumulation of lipofuscin in the RPE and (2) photoreceptor degeneration over a morphologically intact RPE layer (Kay et al., 2012; Tsang and Sharma, 2018, Lima de Carvalho et al., 2019). A limited number of previous reports analyzed the histopathology of BD donor eyes (Frangieh et al., 1982; Weingeist et al., 1982, O’Gorman et al., 1988; Mullins et al., 2005, Bakall et al., 2007; Mullins et al., 2007). Here, we describe and compare the histology and molecular pathology in donor eyes from two patients with BD caused by c.886A > C (p.Asn296His) and c.602T > C (p.Ile201Thr) *BEST1* variants to provide insight into the pathophysiology of the disease. This is the first study of adult postmortem donor eyes from patients with BD due to these specific mutations.

## Materials and Methods

### Donor Eye Acquisition, Imaging, and Genotyping

Postmortem eyes obtained from the Cole Eye Institute Eye Tissue Repository through the Foundation Fighting Blindness (FFB) Eye Donor Program (Columbia, MD). Eyes from BD donors (FFB# 928 and 458) were enucleated and fixed in 4% paraformaldehyde (PF) and 0.5% glutaraldehyde (GA) in D-PBS 12 and 25 h postmortem. Donors were an 85-year-old female and a 65-year-old male. Normal postmortem donor eyes from an anonymous 65- and 95-year-old woman and an 88-year-old male were fixed similarly within 4 and 18 h postmortem (FFB# 696, 784, and 789).

Eyes were cut through the ora serrata, transferred to a plexiglass chamber filled with D-PBS, and imaged by Spectral Domain-Optical Coherence Tomography (SD-OCT) and confocal scanning laser ophthalmoscopy (cSLO) as previously described (Bagheri et al., 2012). For the SD-OCT images, a single telecentric objective lens was employed to collect 5 × 5 mm and 10 × 10 mm FOV of the posterior pole using the following scan parameters: (1) 5-mm linear scan of the horizontal meridian through the optic nerve and fovea @ 1000 A-scans/B-scan, (2) 10-mm linear scan of the horizontal meridian through the optic nerve and fovea @ 1000 A-scans/B-scan, (3) 5-mm^2^ volume scan of the posterior pole @ 500 B-scans/volume × 250 A-scans/B-scan, and (4) 10-mm^2^ volume scan of the posterior pole @ 500 B-scans/volume × 250 A-scans/B-scan. SLO images were collected using a model HRA2 confocal scanning laser ophthalmoscope (Heidelberg Engineering, Inc.). The HRA2 was rotated 90^*o*^ so that the scan direction was perpendicular to the table surface. The system was operated in high-resolution mode, which provides an image pixel format of 1536 × 1536 when used with a 55° wide-field objective lens. SLO images of the posterior pole were collected using infrared reflectance (SLO-IR), infrared dark field (SLO-IRDF), autofluorescence (SLO-AF), and red-free dark field (SLO-RFDF) imaging modes at field of view (FOV) settings of 55°, 35°, and 25°.

Sequence analysis of the entire *BEST1* coding region was performed and reported by Dr. Edwin Stone (Lotery et al., 2000). DNA analysis of donor 1 detected a c.886A > C (p.Asn296His) *BEST1* pathogenic variant and a c.602T > C (p.Ile201Thr) *BEST1* pathogenic variant in donor 2; both mutations were heterozygous. The clinical evaluation of donor 1 was carried out at the University of Illinois with the approval of the Institutional Review Board (IRB) at the University of Illinois Medical Center.

### Retina Histology

Fragments of retina–RPE–choroid were cut from the perifovea and periphery. Tissue fragments were further fixed by immersion in 2.5% GA in 0.1 M cacodylate buffer, post-fixed with 1% osmium tetroxide for 45 min on ice, sequentially dehydrated in ethanol, and embedded in Epon as previously described (Bonilha et al., 2015). Toluidine blue-stained sections were photographed with a Zeiss AxioImager. Z1 light microscope equipped with an MRc5 camera (Carl Zeiss AG, Oberkochen, Germany).

### Immunohistopathology of Photoreceptors and RPE Layers

Another set of tissue fragments was fixed by immersion in 4% PF in D-PBS where they remained overnight at 4°C and then quenched with 50 mM NH_4_Cl in D-PBS for 1 h at 4°C followed by changes to 10% (1 h) and 20% sucrose (overnight) made in the same buffer and finally a mix of 20% sucrose and Tissue-Tek “4583” (Miles Inc., Elkhart, IN). Finally, samples were transferred to a small cassette filled with the same sucrose and Tissue-Tek mix and frozen. Cryosections (10 μm) were collected on an HM 505E cryostat (Microm, Walldorf, Germany) equipped with a CryoJane Tape-Transfer system (Leica, St. Louis, MO). No perifoveal tissue from donor 2 was available for this analysis.

Autofluorescence of unlabeled cryosections was performed and analyzed using epifluorescence in the green channel (FITC filter: 490 nm excitation/519 nm emission) and red channel (TRITC filter: 550 nm excitation/570 nm emission). Autofluorescence was overlaid on differential interference contrast (DIC) images.

Cryosections were blocked in D-PBS supplemented with 2% BSA and 0.2% TX100 (D-PBS/BSA/TX) for 30 min and incubated with the following antibodies: GFAP (mouse, ab10062, 1:400, Abcam, Cambridge, MA), rhodopsin (mouse, ab5417, 1:1000, Abcam), MCT3 (rabbit, 1:100, a gift from Dr. N. Philp, Philadelphia University), EBP50 (rabbit, 1:200, Thermo Fisher Scientific, Waltham, MA), bestrophin-1 (NB300-164, mouse, E6-6,1:50, Novus Biologicals, Littleton, CO), and red/green opsin (AB5405, rabbit, 1:600, Millipore Sigma-Aldrich, Billerica, MA), in PBS/BSA/TX overnight at 4°C. Sections were then labeled with secondary antibodies conjugated with Alexa Fluor 488 and 594 (Molecular Probes, 1:1000) for 45 min at room temperature. Cell nuclei were labeled with TO-PRO^®^-3 iodide (Thermo Fisher). Sections were also labeled with PNA-Alexa488 (Thermo Fisher Scientific, 1:100) and WGA-Alexa594 (Thermo Fisher Scientific, 1:500). Images were acquired using a Leica laser scanning confocal microscope (TCS-SP8, Leica, Exton, PA) with a series of 0.33 μm xy (en face) optical sections. Microscopic panels were composed using Adobe Photoshop CC (Adobe, San Jose, CA). The perifovea of donor 2 was unavailable for analysis.

## Results

### Clinical Findings

Donor 1 was last seen for a follow-up eye examination in September 1992 at 65 years of age, 20 years before her death. At that time, her visual acuity was correctable to 20/200 in both eyes. The lenses showed trace nuclear sclerosis. Ocular pressures were 18 mmHg in each eye. The fundus examination showed a hypertrophic scar in the right eye (Supplementary Figure 1A, OD), while the left eye showed areas of hypopigmentation within the fovea (Supplementary Figure 1A, OS). The patient reported a blurred vision in the right eye; this eye displayed a + 2 1/2 anterior cortical change. The left eye showed a + 1 anterior cortical opacity. Her visual field showed bilateral central scotomas. The donor was on blood pressure medication and a water pill. Her systemic health was negative for other major medical problems.

Due to the retrospective nature of this analysis, historical clinical records were unable to be obtained for donor 2.

### Fundus Macroscopy and Histopathology of *BEST1* Mutations

**FIGURE 1:**
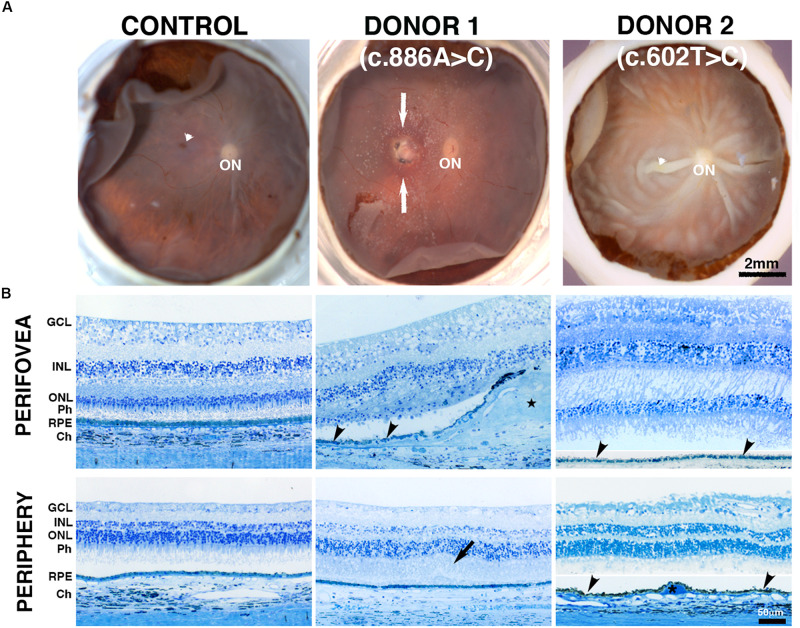
Impact of *BEST1* pathogenic variants on gross pathology and retinal morphology compared to an unaffected, age-matched control eye. **(A)** The macroscopic fundus image shows a control eye free of any pathology. Donor 1 (c.886A > C, p.Asn296His, an 85-year-old donor) displayed a visible macular lesion (white arrows) while donor 2 (c.602T > C, p.Ile201Thr, a 65-year-old donor) did not display any obvious retinal lesion; however, this donor displayed significant postmortem fixation artifacts (areas of retinal detachment). Visible fovea is indicated by white arrowhead, optic nerve head = ON. **(B)** Histology of a control retina (a 95-year-old donor) in the perifovea and periphery displayed typical characteristics including structured lamina consisting of retinal cells. Donor 1 perifovea shows a fibrovascular scar present between the Bruch’s membrane and the retina (star), accompanied by thin patchy RPE (black arrowhead) and inter photoreceptor matrix edema (black arrow); asterisk = drusen. GCL = ganglion cell layer; INL = inner nuclear layer; ONL = outer nuclear layer; POS = photoreceptor outer segments; RPE = retinal pigment epithelium, choroid (Ch). In the periphery, both donors 1 and 2 displayed a distinct GCL, INL, ONL, RPE, and Ch. Scale bar: A = 2 mm (all low-magnification images) and Scale bar B = 50 μm (all images).

Pathogenic variants in *BEST1* affect the function of bestrophin-1 and disrupt the ion transport by the RPE, resulting in the accumulation of fluid between the RPE and the photoreceptors (Singh et al., 2013, Marmorstein et al., 2015, Milenkovic et al., 2018; Nachtigal et al., 2020). This change in subretinal fluid likely results in separation of the neural retina from the RPE and the observation of the typical yellow yolk-like macular lesion upon fundus examination (MacDonald et al., 1993-2020). Images from donor 1 [c.886A > C (p.Asn296His) variant] showed an irregular whitish macular lesion with areas of hyperpigmentation that was more visible by visible light fundus macroscopy (Figure 1A) than either IRDF-cSLO or BAF-cSLO (Supplementary Figure 2A). Donor 2 [c.602T > C (p.Ile201Thr) variant] was absent of any obvious retinal lesions (Figure 1A). Histologically, an extensive fibrovascular scar was present in the perifovea of donor 1 (Figure 1B, star) via fundus microscopy. Immediately above the scar, the retina’s outer nuclear layer was absent; this area also displayed thin RPE (Figure 1B) with no photoreceptors and contained some choroidal vessels. Adjacent to the scar, the retina’s outer nuclear layer was reduced to a single discontinuous row of photoreceptor cell nuclei; the inner segments of surviving photoreceptor cells were shortened. In contrast, the perifovea of donor 2 had distinct ganglion cell layer (GCL), inner nuclear layer (INL), outer nuclear layer (ONL), and RPE (Figure 1B). In the periphery, both donor 1 and donor 2 displayed distinct GCL, INL, ONL, RPE, and choroid (Ch). Donor 1 also displayed edema of the interphotoreceptor matrix (Figure 1B, arrow).

**FIGURE 2:**
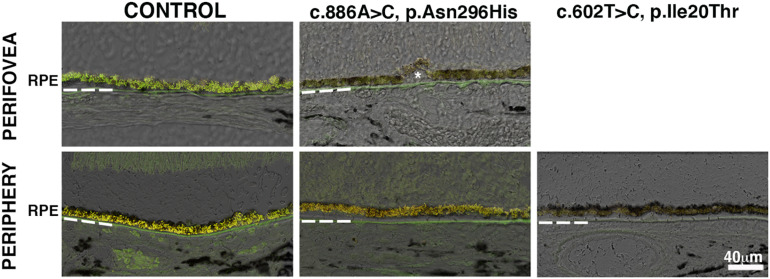
Impact of *BEST1* pathogenic variants in RPE autofluorescent granules. Cryosections obtained from the BD donors and a 95-year-old control were observed using the green channel (FITC filter) and red channel (TRITC filter). Autofluorescence was overlaid on differential interference contrast (DIC) images. Bruch’s membrane is indicated by hashed white line, asterisk = drusen. Scale bar = 40 μm (all images).

The frequently observed vitelliform lesion in BD patients localizes to the macula’s subretinal space and contains fluid and lipofuscin. Lipofuscin is a long-lived intracellular inclusion body, lipid- and bisretinoids-rich, and autofluorescent material that progressively accumulates in the RPE during aging and pathological conditions as BD (Ng et al., 2008; Sparrow et al., 2010, 2012). We compared the relative amount of autofluorescence in the RPE of control and BD eyes. RPE in the perifovea and periphery of donor 1 showed a substantial decrease of autofluorescence compared to RPE in the control eyes (Figure 2). Also, in the periphery of donor 2, the RPE showed a paucity of autofluorescence compared to the control RPE.

### Photoreceptor Pathology of *BEST1* Mutations

**FIGURE 3:**
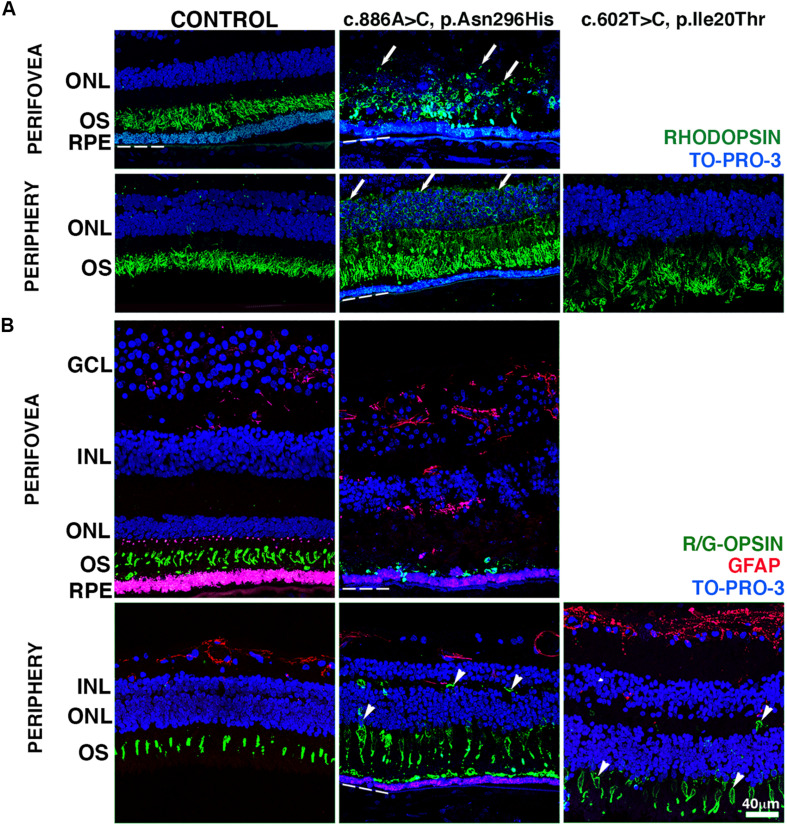
Impact of *BEST1* pathogenic variants in photoreceptors. **(A)** Cryosections obtained from the BD donors and a 65-year-old control were labeled with antibodies specific to rhodopsin (green), while cell nuclei were labeled with TO-PRO-3 (blue). **(B)** Cryosections were also labeled with antibodies specific to red/green cone opsin (green) and GFAP (red), while cell nuclei have been labeled with TO-PRO-3 (blue). Arrow = abnormal distribution of rhodopsin into cell body; arrowheads = abnormal distribution of red/green cone opsin into the cell body. Bruch’s membrane is indicated by the hashed white line. Scale bar = 40 μm (all images).

The RPE–photoreceptor interface is an area of fundamental importance for supporting the proper retinal function. To gain insight, we carried out IHC evaluation of the BD retinas using a set of markers with known expression in the rod and cone outer segments. The distribution of rhodopsin was restricted to the outer segments of the control donor in both the perifovea and periphery (Figure 3A). In the perifovea of donor 1, rhodopsin labeling displayed a circular pattern close to the RPE surface, with a few cellular projections being observed in the outer plexiform layer. Redistribution of rhodopsin throughout the whole photoreceptor cell (Figure 3A, arrows) was observed in the periphery of donor 1. In the periphery of donor 2, rhodopsin labeling was overall decreased; outer segments were disorganized, and rhodopsin was distributed in the inner segments (Figure 3A).

We then investigated the distribution of cone photoreceptors in retinas harboring *BEST1* variants using red/green cone opsin labeling. In the control retina, red/green cone opsin was distributed along with the outer segments in the perifovea and periphery (Figure 3B, green). Red/green opsin-labeled cells were mostly absent in the retina adjacent to the fibrovascular scar of donor 1. Abnormal distribution of the red/green opsins throughout the entire cone cell body was observed in the periphery of donors 1 and 2 (Figure 3B, arrowheads). Moreover, Müller cells, labeled with GFAP antibodies, had undergone extensive activation throughout the retina. Their hypertrophic processes were observed in the periphery of donor 2 and perifovea of donor 1 (Figure 3B, red) when compared to the control retina. In the subretinal space, the interphotoreceptor matrix (IPM) surrounding the inner and outer segments of the cone (Supplementary Figure 3, green) and rod (Supplementary Figure 3, red) photoreceptors was analyzed in both control and BD donor retinas labeled with PNA and WGA lectins. In the control retina, PNA (green) bound to the extracellular matrix sheaths of the cone photoreceptor inner and outer segments while WGA (red) bound to the extracellular matrix sheaths of the rod photoreceptor inner and outer segments in both the perifovea and periphery. In the perifovea of donor 1, PNA and WGA labeling were observed dispersed through the photoreceptor inner and outer segments. In the periphery, PNA and WGA labeling colocalized in the outer segments. In the periphery of donor 1, PNA and WGA were also dispersed through the photoreceptor inner and outer segments; a visible decrease in PNA labeling was observed. PNA and WGA labeling evidenced edema of the IPM in both the perifovea and periphery of donor 1 (Supplementary Figure 3, arrows). In the periphery of donor 2, PNA and WGA labeling were visibly decreased, but restricted to the outer segments.

### RPE Pathology of *BEST1* Mutations

**FIGURE 4:**
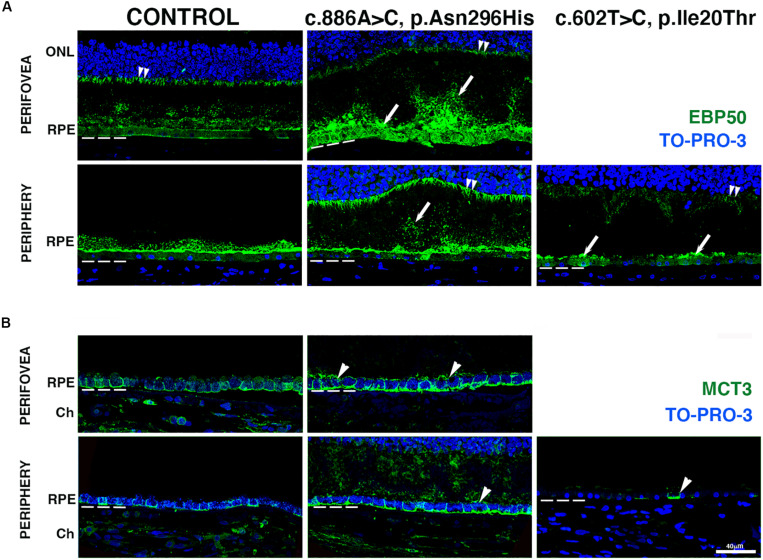
Impact of *BEST1* pathogenic variants in gross pathology and RPE. **(A)** Cryosections obtained from the BD donors and an 88-year-old control were labeled with antibodies specific to EBP50 (green), while cell nuclei were labeled with TO-PRO-3 (blue). **(B)** Cryosections were also labeled with antibodies specific to MCT3 (green), while cell nuclei have been labeled with TO-PRO-3 (blue). Bruch’s membrane is indicated by the hashed white line. Arrow = abnormal RPE apical microvilli; arrowheads = mislocalized apical RPE distribution of MCT3; double arrowheads = Muller cell apical processes. Scale bar = 40 μm (all images).

To gain insight into the effects of the *BEST1* variants on RPE morphology, we carried out IHC evaluation of the BD retinas using markers known to be expressed at either the RPE apical or basolateral surfaces. The distribution of ERM (ezrin, radixin, and moesin)-binding phosphoprotein of 50 kDa (EBP50), a protein that links apical transporters to ezrin and the actin cytoskeleton, was observed at the RPE apical microvilli in both the perifovea and periphery of the control retina; a minor presence was also observed in the basal surface of the cells as previously described (Bonilha and Rodriguez-Boulan, 2001; Nawrot et al., 2004). EBP50 also labeled Müller cell apical processes (Figure 4A, double arrowheads). In the perifovea of donor 1, EBP50 labeling significantly increased; the RPE apical microvilli were highly disorganized and seemed to form patchy structures of different lengths and thickness projecting into the photoreceptors. In the periphery of donor 1, similar but lessened EBP50 apical distribution was observed; a correspondent presence of basal punctate structures was observed (Figure 4A, arrows). In the periphery of donor 2, overall decreased EBP50 labeling of the apical RPE surface was observed. Deformed (domed shape) Müller cell apical processes were visible in all BD donor retinas due to the photoreceptor alterations.

We also investigated the distribution of monocarboxylate transporter 3 (MCT3), an RPE basolateral transporter (Philp et al., 2003). In both the perifovea and periphery of control retinas, MCT3 was localized to the RPE basolateral surface (Figure 4B). In the perifovea of donor 1, MCT3 was observed in all aspects of the RPE membrane (Figure 4B, arrowheads) with increased labeling density relative to control. In the periphery of donor 1, MCT3 was mostly confined to the basolateral surface; however, it was also observed in the RPE apical microvilli and extended up to the photoreceptor nuclei (Figure 4B). In the periphery of donor 2, MCT3 was notably absent, with just a few cells labeled (Figure 4B, arrowhead).

**FIGURE 5:**
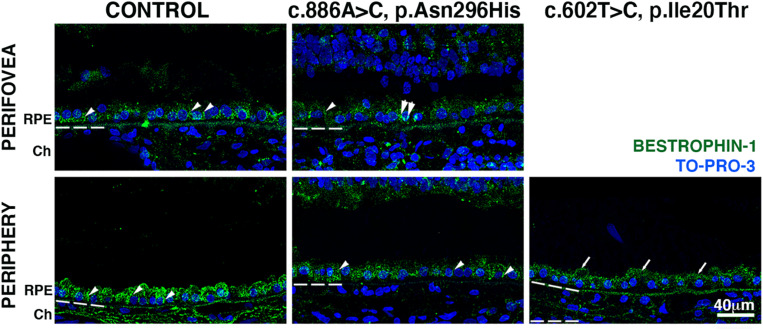
Impact of *BEST1* pathogenic variants in bestrophin-1 RPE localization. Cryosections obtained from the BD donors and an 88-year-old control were labeled with antibodies specific to bestrophin-1 (green), while cell nuclei have been labeled with TO-PRO-3 (blue). Bruch’s membrane is indicated by the hashed white line. Arrow = mislocalized apical RPE distribution of bestrophin-1; arrowheads = basolateral RPE distribution of bestrophin-1; double arrowheads = intracellular bestrophin-1. Scale bar = 40 μm (all images).

Finally, we investigated bestrophin-1 distribution in control and BD eyes (Figure 5). Immunolabeled bestrophin-1 was more highly expressed in the periphery than in the perifovea of control samples, as previously reported (Mullins et al., 2007). In the perifovea of donor 1, decreased labeling of the RPE was observed; there were a few cytoplasmic punctated structures (Figure 5, arrows). In the periphery, labeling was present but diminished and still localized mostly to the basal surface and cytoplasm. In the periphery, of donor 2, overall bestrophin-1 labeling was also decreased, and the protein localized mostly to the apical surface.

## Discussion

BD is an inherited macular degeneration with variable penetrance and expressivity characterized by the loss of central vision, accompanied by the inability to perceive colors and resolve detail. A few previous studies reported the histopathology of donor eyes harboring known *BEST1* pathogenic variants p.Thr6Arg (Mullins et al., 2007), p.Trp93Cys (Bakall et al., 2007), and p.Tyr227Asn (Mullins et al., 2005, 2007). Here, we report the retinal findings from donors with a clinical diagnosis of BD harboring c.886A > C (p.Asn296His) (donor 1) or c.602T > C (p.Ile201Thr) (donor 2) variants in *BEST1* gene; the main findings previously described and in the present study are summarized in Table 1. These variants have been reported previously (Lotery et al., 2000), but to our knowledge, this is the first report on histopathological findings in the retinas from donors with these variants.

**TABLE 1:** Main retinal features observed in retinas of BD donors with known bestrophin-1 mutations.

**Manuscript**	**Histological findings (Macula-Perifovea)**	**Immunohistochemical findings (Macula-Perifovea)**	**Histological findings (Perimacula-Periphery)**	**Immunohistochemical findings (Perimacula-Periphery)**
Mullins et al., 2005 (**Y227N** mutation)	– ONL attenuation and a region of severe photoreceptor degeneration resembling a scar and preservation of viable choriocapillaris	– negative GFAP labeling; – positive fibrinogen labeling; – positive BCIP/NBT kit (to detect vessels);	– remarkable degree of outer nuclear layer attenuation; – multiple drusen and areas of RPE detachment; – significant accumulation of basal laminar deposits; – occasional areas of RPE and photoreceptor atrophy	– typical GFAP labeling; – positive rhodopsin labeling; – bestrophin-1 labeling along the apical membrane, cytosol, and the basolateral membrane; – no obvious increase in the size, fluorescence intensity, or number of lipofuscin granules;
Mullins et al., 2007- (**T6R** mutation)	– disciform scarring (with RPE and ONL degeneration); – photoreceptor dropout and gliosis; – presence of “ghost” vessels;		– normal histology; – some peripheral drusen; – focal loss of inner and outer segments and ONL attenuation;	– increased GFAP labeling in areas of photoreceptor loss; – decreased lipofuscin accumulation;
Mullins et al., 2007- (**Y227N** mutation)	– disciform scarring; – photoreceptor dropout and gliosis; – RPE degeneration; – presence of “ghost” vessels;			
Bakall et al., 2007 (**W93C** mutation)	– localized regions of severe retinal degeneration with all retinal layers affected		– large serous retinal detachment; – focal loss of RPE cell; – well preserved retinal layering;	– bestrophin-1 staining localized throughout the RPE; – classical lipofuscin granules (the least dense fraction, in sucrose gradient) was either not present or significantly diminished, however, granules in fractions of higher density were increased;
Present study [**c.886A > C (p.Asn296His**) mutation]	– fibrovascular scar; – ONL was absent; – thin RPE;	– substantial decrease of autofluorescent granules; – rhodopsin labeling displayed a circular pattern close to the RPE surface, with a few cellular projections being observed in the ONL; – red/green opsin labeling significantly decreased; – increased GFAP labeling; – EBP50 labeling significantly increased (highly disorganized RPE apical microvilli seemed to form patchy structures of different lengths and thickness); – MCT3 labeling increased (in all aspects of the RPE membrane); – decreased bestrophin-1 labeling (cytoplasmic punctated); – PNA labeling dispersed through inner and outer segments; – WGA labeling dispersed through inner and outer segments);	– distinct GCL, INL, ONL, RPE and choroid; – edema of the interphotoreceptor matrix	– substantial decrease of autofluorescent granules; – rhodopsin throughout the whole photoreceptor cell; – red/green opsin labeling throughout the entire cone cell body; – EBP50 labeling increased (disorganized RPE apical microvilli seemed to form patchy structures of different lengths and thickness); – MCT3 labeling mostly confined to the basolateral surface (apical in a few cells); – decreased bestrophin-1 labeling (mostly basolateral and cytoplasmic); – visibly decreased PNA labeling (dispersed through inner and outer segments); – WGA labeling dispersed through inner and outer segments;
Present study [**c.602T > C (p.Ile201Thr**) mutation]	– distinct GCL, INL, ONL and RPE;	NOT ANALYZED	– distinct GCL, INL, ONL, RPE and choroid;	– paucity of autofluorescent granules; – overall rhodopsin labeling decreased; – red/green opsin labeling throughout the entire cone cell body; – increased GFAP labeling; – MCT3 labeling notably absent (a few cells labeled); – decreased bestrophin-1 labeling (mostly apical and cytoplasmic); – PNA labeling visibly decreased (outer segments); – WGA labeling visibly decreased (outer segments).

In our study, donor 1 [c.886A > C (p.Asn296His) *BEST1* variant] displayed a central macular lesion that was visible by fundus macroscopy. Immunohistological analysis of this tissue revealed that rod photoreceptors were less affected than cones. In the perifovea, rhodopsin labeling displayed a circular pattern close to the RPE surface, with a few cellular projections being observed in the outer plexiform layer. At the same time, red/green opsin-labeled cones were mostly absent. Although the RPE monolayer was morphologically intact, the distribution of plasma membrane proteins was significantly decreased in the periphery; the apical microvilli labeled by EBP50 could be observed to re-organize into patchy areas of variable length with several short and enlarged microvilli and increased basolateral and cytoplasmic distribution while the transporter MCT3 was distributed in both the apical and basolateral membranes. Similar but lessened changes were observed in the periphery of this donor.

The fundus macroscopy of the retina of donor 2 [c.602T > C (p.Ile201Thr) *BEST1* variant] did not display any retinal lesion. However, it did display a substantial fixation artifact in the form of several small areas of retinal detachment. Immunohistological analysis of this tissue revealed that rhodopsin labeling was restricted to the rod outer segments but reduced in expression and substantially disorganized. While cone opsins were distributed throughout the whole cell in the periphery, the distribution of RPE plasma membrane proteins was significantly decreased in the periphery; EBP50 distribution was similar to that observed in control samples, but MCT3 was mostly absent from the RPE.

Müller cells upregulate the expression of the intermediate filament GFAP in response to retinal diseases and injuries (Bringmann et al., 2006). Older retinas frequently have only isolated glial cells overlying large blood vessels, whereas more extensive membranes are associated with disease (Edwards et al., 2016). Based on this previous report, we observed strong GFAP labeling associated with the control samples’ blood vessels. Increased GFAP labeling in the periphery of donor 2 and perifovea of donor 1 was observed. A previous study has shown GFAP labeling outside the scar and in the interface between the scar and Bruch’s membrane of eyes with BD donor possessing a p.Tyr227Asn mutation (Mullins et al., 2005).

Prior reports indicated that BD is characterized histopathologically by accumulating abnormal lipofuscin in the RPE (O’Gorman et al., 1988; Mullins et al., 2007). Although autofluorescent granules were detected in the RPE cytoplasm, their presence decreased compared to control samples. Our results agree with a previous study that reported a significant decrease in classical lipofuscin granules in the BD donor eyes harboring a p.Trp93Cys pathogenic variant in *BEST1* (Bakall et al., 2007). Our observations could be a direct result of the *BEST1* mutations and their consequences in the retinal physiology. Alternatively, our observations could be related to the disease stage or prolonged exposure to light during eye processing.

Because a primary defect in RPE causes BD, we analyzed the distribution of RPE markers. EBP50 is a PDZ-scaffold protein initially identified as an organizer and modulator of transporters and channels and links apical transporters such as the cystic fibrosis transmembrane conductance regulator (CFTR), the kidney proximal tubule Na + /H + exchanger (NHE3), and the β2-adrenergic receptor to ezrin and the actin cytoskeleton in epithelial microvilli. In the RPE apical microvilli, EBP50 binds to ezrin and the retinoid-binding protein CRALBP through different domains (Bonilha and Rodriguez-Boulan, 2001; Nawrot et al., 2004). In the present study, EBP50 labeling significantly increased in both the perifovea and periphery of donor 1, but it decreased in the apical RPE surface of the periphery of donor 2. EBP50 is upregulated during RPE aging (Gu et al., 2012) and cellular senescence (Althubiti et al., 2014). Moreover, EBP50 is upregulated in diverse cancers where its level of expression correlates with aggressive stage and poor prognosis (Vaquero et al., 2017).

The presence of MCT3, an RPE basolateral transporter (Philp et al., 2003) with an important role in regulating pH and lactate concentrations, was also analyzed. In the present study, MCT3 was observed in all aspects of the RPE membrane of donor 1; in the periphery, MCT3 was mostly confined to the basolateral surface; however, it was also observed in the RPE apical microvilli. In the periphery of donor 2, MCT3 was notably absent, with just a few cells labeled. Mutations in the MCT3 gene have not been linked to retinal disease; however, a previous report described that wounding of RPE monolayers resulted in the dedifferentiation of the cells at the edge of the wound in association with loss of MCT3 (Gallagher-Colombo et al., 2010). The RPE performs nursing functions that regulate and determine the health of the photoreceptors. All these functions rely on the presence of diverse plasma membrane transporters and receptors present either in the apical or in the basolateral membrane domains of RPE. Alterations in the expression or targeting of RPE proteins such as EBP50 and MCT3 would be expected to have a severe impact on the chemical composition of the subretinal space and on photoreceptors function and are thus related to the photoreceptor changes observed in our samples.

The gene responsible for BD is the *BEST1* gene, which encodes bestrophin-1, a transmembrane channel localized to the RPE basolateral plasma membrane. Bestrophin-1 has been extensively studied and described as a multifunctional protein implicated in mediating the flow of ions across the RPE, regulating calcium signaling and cell volume, and modulating the subretinal space milieu (Rosenthal et al., 2006; Hartzell et al., 2008, Neussert et al., 2010; Kane Dickson et al., 2014, Strauss et al., 2014; Yang et al., 2014, Milenkovic et al., 2015; Guziewicz et al., 2018). However, its multifaceted nature and complex interactions with photoreceptors in health and disease remain unsolved (Guziewicz et al., 2017). Here, we detected bestrophin-1 immunohistochemical labeling to the basal surface of the RPE in the periphery of the eye from donor 1 [c.886A > C (p.Asn296His) *BEST1* variant]. In contrast, it was mostly present in the RPE apical surface in the periphery of the eye from donor 2 [c.602T > C (p.Ile201Thr) *BEST1* variant]. The c.704T > C; p.(V235A) *BEST1* mutation was previously reported to be mislocalized at least in part to the apical surface of hiPSC-RPEs from an autosomal dominant vitreoretinochoroidopathy patient (Carter et al., 2016). Our observations suggest that proper bestrophin-1 localization is mutation-dependent. Moreover, our data suggest that BD results from bestrophin-1 dysfunction and its consequences in the RPE function.

Significant insight into the BD pathological mechanisms has been obtained from recent studies employing stem cells for disease modeling since RPE can be readily produced and purified (Singh et al., 2013; Domingo-Prim et al., 2019, Nachtigal et al., 2020). Specifically, human iPS cell (hiPSC)-RPE who harbored p.Ala146Lys or p.Asn296His mutations in *BEST1* reported appropriately polarized distribution of plasma membrane proteins and displayed typical RPE features including apical microvilli, intracellular pigment granules, and uniformly expressed tight junction protein ZO-1 in tight-junctional complexes but displayed decreased net fluid transport and delayed degradation of photoreceptor outer segments associated with increased oxidative stress (Singh et al., 2013). Remarkably, the localization and distribution of bestrophin-1 were similar in the control and BD hiPSC-RPE cells, suggesting that BD most likely resulted from bestrophin-1 dysfunction. Significantly higher autoflourescence levels were detected in BD hiPSC-RPE.

The data presented here provide new insights into the pathology and disease manifestation caused by c.886A > C (p.Asn296His) and c.602T > C (p.Ile201Thr) *BEST1* pathogenic variants. Although the sample size is limited, these two examples suggest that different variants in the *BEST1* gene can result in substantially different diagnostic imaging phenotypes. The different RPE phenotypes observed in the donor eyes and in the BD hiPSC-RPE may result from how the individual variants affect bestrophin-1 structure and function, and how these consequently modulate the subretinal space and photoreceptors. Alternatively, the presence and interaction of one or more modifier genes with *BEST1* may affect the expressivity of the mutation and their manifestation into BD, as previously proposed (Mullins et al., 2007).

Presently, there is no treatment available to treat BD; thus, a better understanding of *BEST1*-related pathogenesis may help to define therapeutic targets. Our results suggest that although BD etiology remains poorly understood, further efforts to understand the unique pathogenesis of each BEST1 mutation are warranted. Only with the full understanding of the cellular and tissue effects on the pathologies is a targeted and efficient therapeutic approach plausible and promises to be successful in the long term. The lower level of bestrophin-1 protein found in both RPE cells does suggest that increasing the levels of this protein, through gene augmentation therapy or by rescuing mutant Best1 from proteasomal degradation, may be a viable means of preventing vision loss in BD. This study resulted in tangible improvements in our understanding of BD pathology. However, it is a snapshot of the BD pathology caused by the BEST variants. Moreover, our study was limited by the lack of clinical data and by the unavailability of perifoveal tissue from donor 2 to be analyzed by immunohistochemistry.

## Data Availability Statement

The datasets generated for this study are available on request to the corresponding author.

## Ethics Statement

All procedures in this study adhered to the tenets of the Declaration of Helsinki regarding research involving human tissue and were approved by the Institutional Review Boards of the Cleveland Clinic (IRB14-057).

## Author Contributions

VLB and JGH performed the conceptualization. GAF performed clinical input. VLB, BAB, and MJD performed experimental input. VLB, GAF, SAH, and JGH provided resources. All authors have read, reviewed, edited, and agreed to the published version of the manuscript.

## Conflict of Interest

The authors declare that the research was conducted in the absence of any commercial or financial relationships that could be construed as a potential conflict of interest.
