# Supplementary material for: Cellular Changes in Retinas From Patients With BEST1 Mutations
Source: Front Cell Dev Biol. 2020 Oct 14;8:573330. doi: 10.3389/fcell.2020.573330 (PMC7591587; doi:10.3389/fcell.2020.573330)
Supplement: Supplementary file 1 [file Data_Sheet_1.docx]

Supplementary Material

## Supplementary Figures

_
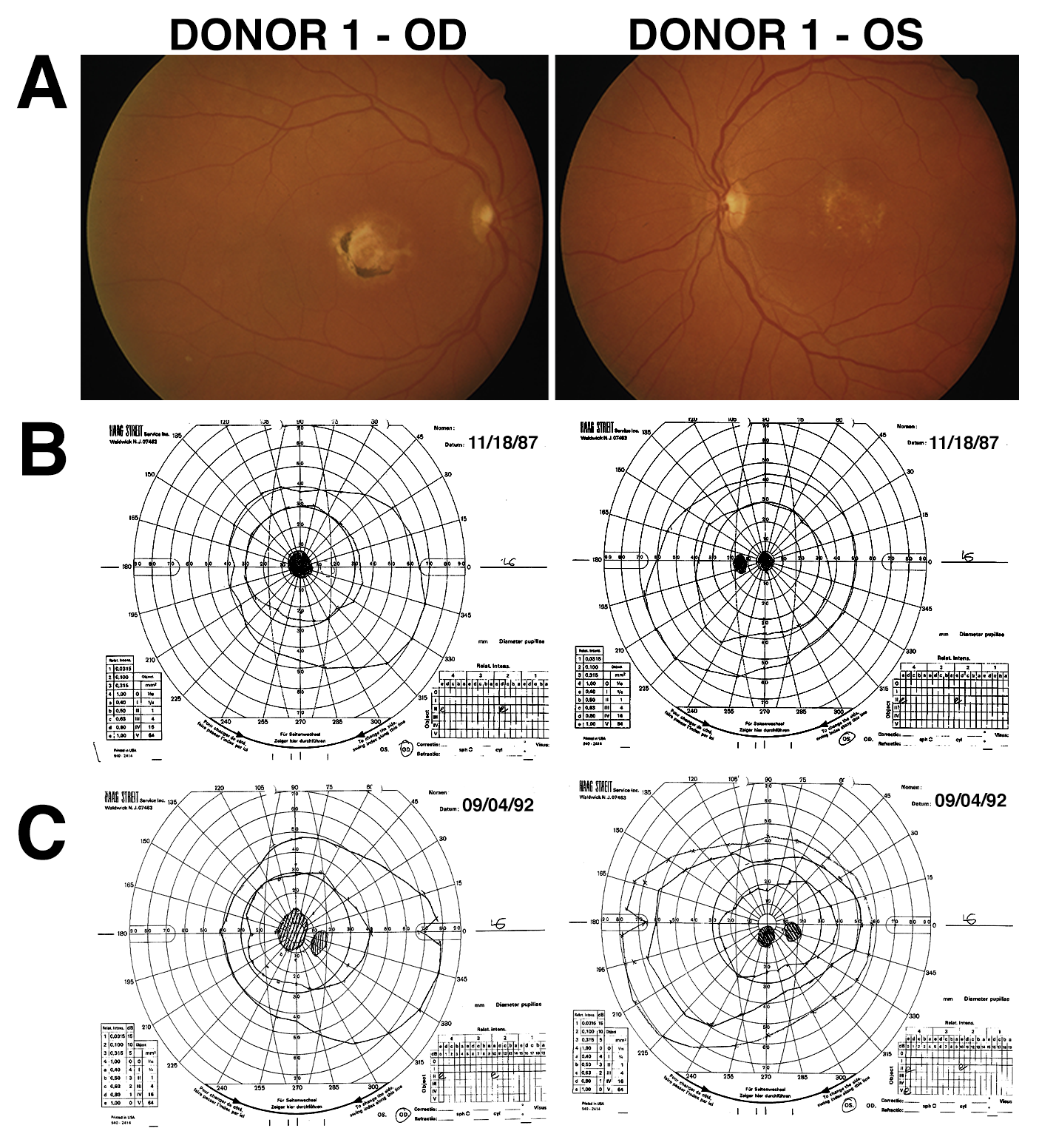
_

**Supplementary Figure 1.** Findings of the BD Donor 1 eye harboring the c.886A>C (p.Asn296His) *BEST1* mutation. **(A)** Fundus photograph OD and OS at age 65 years. (B) Goldmann visual fields to an II-2e white test light at age 60. (C) Goldmann visual fields to an II-2e white test light at age 65.


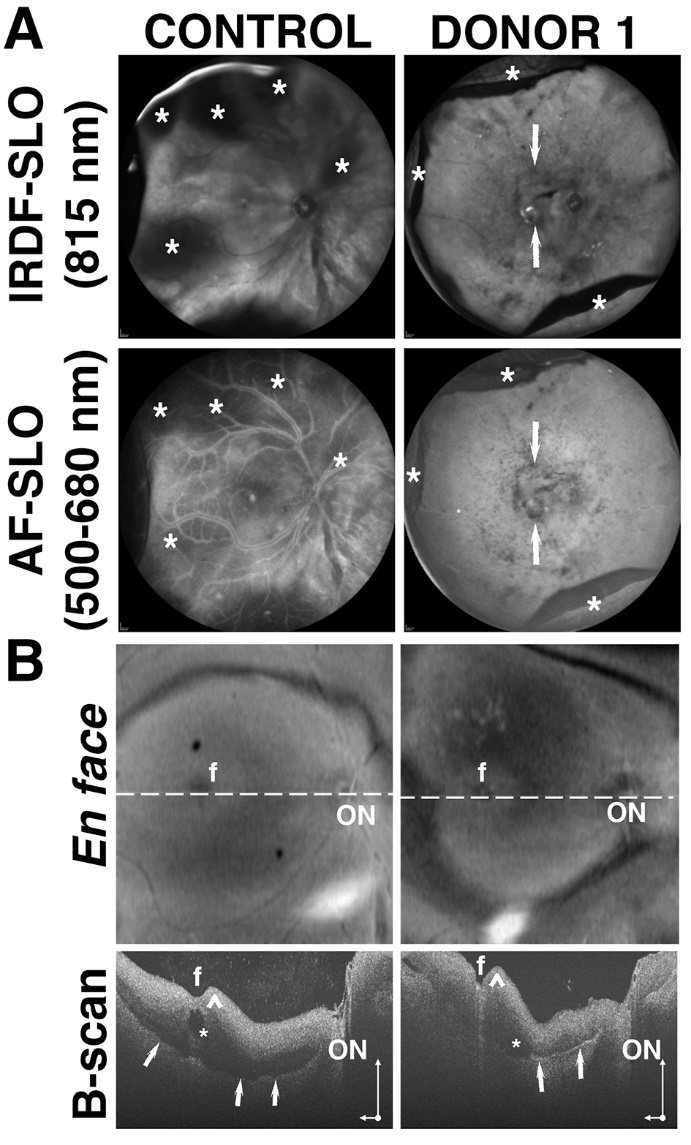


**Supplementary Figure 2.** Ex-vivo imaging of the BD Donor 1 eye harboring the c.886A>C (p.Asn296His) *BEST1* mutation relative to a normal control eye. **(A)** Posterior globes were imaged by confocal SLO using a model HRA2 (Heidelberg Engineering). All eyes showed areas of post-mortem artefact (detached retina; *) due to fixation and processing. All imaging modalities indicated that the control eye was free of retinal pathology. In contrast, images from the donor revealed a prominent macular lesion by IRDF-SLO or BAF-SLO. BAF-SLO showed a bright, autofluorescent fundus which lacked typical detail (absence of a macula lutea) and appeared more homogeneous than the control eye). No SLO or OCT images are available for donor 2. **(B)** Spectral Domain-OCT images were collected using the Model SDOIS system (Bioptigen, Inc.) with ~7x7mm field of view. The control eyes had intact retinas that were still adherent and congruent with the RPE-choroid complex and showed evidence of a photoreceptor layer (dark space above arrows in B-scan). In contrast, the B-scan image from the eye of donor 1 lacked a well-defined photoreceptor layer and exhibited a hyper-reflective RPE (arrows) compared to the control eye. Regions of minor retinal detachment (*) and displaced retina (^) were also observed presumably due to fixation and processing. Dashed lines in *en face* image indicates B-scan location, optic nerve (ON), B-scan scale bar is 0.5mm.

**
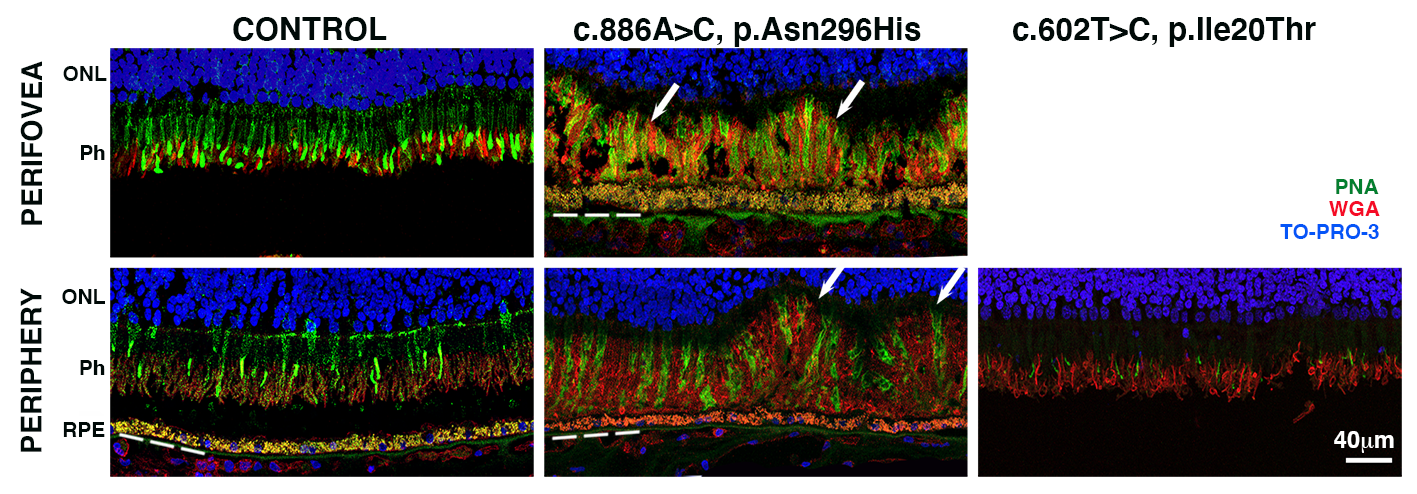
**

**Supplementary Figure 3.** Impact of *BEST1* pathogenic variants in IPM labeling. Cryosections obtained from the BD donors and an 88-year-old control were labeled with lectins specific to cone (green) and rod (red) IPM, while cell nuclei have been labeled with TO-PRO-3 (blue). Bruch’s membrane is indicated by the hashed white line. Arrow = swollen IPM. Scale bar = 40 μm (all images
